# Comparison of Hockey-Stick and Conventional Linear Ultrasound Transducers for Detection of Non-Displaced Dentoalveolar Fractures: An Ex Vivo Paired Comparative Study

**DOI:** 10.3390/tomography12100152

**Published:** 2026-10-21

**Authors:** Murat Mert Atapek, Sedef Ayşe Taşyapan, Elif Yıldızer

**Affiliations:** 1 Faculty of Dentistry, Oral and Maxillofacial Radiology Department, Yeditepe University, Istanbul 34728, Türkiye; 2 Faculty of Dentistry, Oral and Maxillofacial Radiology Department, Istanbul University, Istanbul 34116, Türkiye

**Keywords:** ultrasonography, transducers, fractures, diagnostic imaging, point-of-care systems

## Abstract

**Objectives:** To compare fracture detection rates and observer confidence between a high-frequency hockey-stick transducer and a conventional linear transducer for detecting non-displaced dentoalveolar fractures in a paired ex vivo model. **Materials and Methods:** This paired ex vivo study included 88 experimentally created non-displaced dentoalveolar fracture sites in fresh ovine specimens. Two oral and maxillofacial radiologists independently evaluated all sites with both a 12L3 conventional linear transducer (10–15 MHz) and an 18H5 hockey-stick transducer (10–18 MHz), with transducer order randomized. Cone-beam computed tomography (CBCT) confirmed fracture status. Within-observer paired fracture-detection outcomes were compared using the exact McNemar test, and a cluster-adjusted generalized estimating equation (GEE) analysis accounted for repeated assessments and clustering within ovine heads. Observer confidence was compared using the Wilcoxon signed-rank test. **Results:** Observer 1 detected 76/88 fracture sites (86.4%) with the hockey-stick transducer and 64/88 (72.7%) with the conventional linear transducer (*p* = 0.004). Observer 2 detected 74/88 sites (84.1%) and 62/88 (70.5%), respectively (*p* < 0.001). In the cluster-adjusted GEE analysis, the hockey-stick transducer was associated with higher odds of fracture detection (OR, 2.29; 95% CI, 1.84–2.86; *p* < 0.001). For both observers, confidence scores were higher with the hockey-stick transducer (median [IQR], 4 [3–4] vs. 3 [2–3]; both *p* < 0.001), with Cohen’s dz values of 0.849 and 0.791, respectively. In a separate pre-study calibration set, interobserver agreement was 95% (κ = 0.828; 95% CI, 0.503–1.000). **Conclusions:** In this ex vivo model, the hockey-stick transducer achieved consistently higher fracture-detection rates across both observers and greater observer confidence than the conventional linear transducer. The observed differences may reflect the combined technical and ergonomic characteristics of the two transducers rather than probe geometry alone. Prospective clinical studies including both fractured and non-fractured sites are required before conclusions regarding diagnostic accuracy or clinical superiority can be established.

## Simple Summary

Small fractures around the teeth can be difficult to detect using conventional imaging methods. Ultrasound offers a radiation-free alternative, but the type of probe may influence fracture detection. This experimental study compared two ultrasound probes using 88 artificially created fractures in sheep jaw specimens. The smaller, hockey-stick-shaped probe detected more fractures and provided greater examiner confidence than the conventional linear probe. These findings suggest that probe selection may improve ultrasound-based assessment of dental trauma. However, further studies involving patients and both fractured and non-fractured sites are needed to confirm its clinical usefulness.

## 1. Introduction

Dentoalveolar fractures represent a substantial proportion of maxillofacial injuries, accounting for up to 40–60% of all facial injuries, particularly among children and young adults, and may result in tooth loss, malocclusion, periodontal complications, and impaired oral function if not diagnosed and managed promptly [1,2,3]. Clinical examination is the first step in the evaluation of dentoalveolar trauma; however, pain, soft tissue swelling, hemorrhage, and limited mouth opening frequently reduce its diagnostic accuracy [3,4]. Conventional radiographic techniques, including periapical and panoramic radiography, are routinely used as first-line imaging modalities but may fail to identify subtle or non-displaced fractures because of anatomical superimposition and their two-dimensional nature [5]. Although cone-beam computed tomography (CBCT) provides excellent visualization of dentoalveolar structures and is considered the reference standard for complex injuries, its higher radiation dose, greater cost, and limited accessibility restrict its routine use in many emergency and outpatient settings [5,6].

High-frequency ultrasonography has emerged as a valuable point-of-care imaging modality for the evaluation of superficial maxillofacial injuries because it is non-invasive, free of ionizing radiation, portable, relatively inexpensive, and capable of providing real-time imaging [2,7]. Cortical bone surfaces appear as continuous hyperechoic lines on ultrasonography, whereas fractures are identified as interruptions of this cortical interface [8,9]. Recent advances in high-frequency ultrasound technology have further improved the visualization of superficial osseous structures, expanding the role of ultrasonography in the assessment of maxillofacial trauma [10,11].

Previous studies have demonstrated high diagnostic accuracy of ultrasonography for detecting facial fractures involving the mandible, zygomatic arch, nasal bones, and orbital region [7,11,12,13,14,15]. Nevertheless, relatively little attention has been paid to the influence of transducer design on fracture detection. Probe characteristics such as footprint size, ergonomic configuration, frequency range, and adaptability to the complex contours of the maxillofacial region may substantially affect image acquisition and fracture visualization. The hockey-stick transducer, with its small footprint and angled design, may provide improved access to confined intraoral and curved facial surfaces, whereas conventional linear probes offer a wider field of view but may be less adaptable in anatomically restricted regions [16]. Despite these theoretical advantages, comparative evidence regarding the optimal ultrasound transducer for dentoalveolar fracture detection remains scarce.

A recent periodontal ultrasonography study reported that the hockey-stick transducer demonstrated better measurement reliability than a conventional linear probe, suggesting that transducer geometry may influence ultrasonographic performance [16]. To the best of our knowledge, this is the only previous study directly comparing hockey-stick and conventional linear transducer geometries in the dentomaxillofacial region; it focused on periodontal measurements rather than traumatic fracture detection. Whether these potential advantages translate into improved fracture detection has not yet been investigated under controlled experimental conditions. Identifying the most suitable ultrasound transducer may support the development of ultrasonography as an adjunctive point-of-care imaging tool for dentoalveolar trauma, pending prospective clinical validation.

Therefore, this paired ex vivo study aimed to compare fracture detection rates and observer confidence between a high-frequency hockey-stick transducer and a conventional linear transducer for non-displaced dentoalveolar fractures, using CBCT to confirm fracture status. A secondary aim was to document interobserver agreement in a separate pre-study calibration set. We hypothesized that the hockey-stick transducer would yield a higher fracture detection rate and greater observer confidence because of its smaller footprint and enhanced adaptability to dentoalveolar anatomy.

## 2. Materials and Methods

This paired experimental ex vivo study compared two high-frequency ultrasound transducers for detecting experimentally created non-displaced dentoalveolar fractures. CBCT was used to confirm fracture status before ultrasonographic assessment. Two experienced oral and maxillofacial radiologists evaluated the specimens under standardized imaging conditions, and transducer order was randomized.

### 2.1. Specimens

Fresh sheep heads (*n* = 15), obtained from animals slaughtered for human consumption, were used in this experimental study. Ovine specimens were selected because their dentoalveolar anatomy and cortical bone characteristics closely resemble those of humans, making them a suitable experimental model for simulating dentoalveolar fractures [17]. A total of 88 non-displaced dentoalveolar fracture sites were created under standardized experimental conditions. Fourteen ovine heads contributed six fracture sites each, while the remaining head contributed four fracture sites, yielding a total of 88 fracture sites. Fracture sites were randomly assigned to anatomical regions (maxilla and mandible, anterior and posterior) using a computer-generated randomization sequence before imaging. All specimens underwent CBCT examination prior to ultrasonographic evaluation. Ethical approval was not required because no live animals were involved, and all specimens were obtained from commercial sources after slaughter for food production.

### 2.2. Sample Size Estimation

An a priori sample size calculation was performed using G*Power software (version 3.1.9.7; Düsseldorf, Germany), using a medium standardized effect size (Cohen’s w = 0.30), an alpha level of 0.05, statistical power of 80%, and 1 degree of freedom; this yielded a target of 88 sites. Because the primary comparison ultimately involved paired binary outcomes assessed at the same fracture sites, the exact McNemar test was used for the primary analysis. The original sample-size calculation should therefore be regarded as an approximate planning calculation rather than a McNemar-specific power analysis.

### 2.3. Surgical Procedure

Non-displaced dentoalveolar fractures were experimentally created under standardized conditions using controlled mechanical force applied with dental extraction forceps. For the purposes of this study, a fracture was considered non-displaced when cortical disruption was present without visible displacement of the alveolar segment. All sites included in the primary analysis were confirmed as fractured on CBCT by an experienced oral and maxillofacial radiologist who did not participate in the ultrasonographic assessments. Accordingly, the primary endpoint was fracture detection (sensitivity within fracture-positive sites), rather than full diagnostic accuracy, because non-fractured control sites were not included.

### 2.4. CBCT Acquisition

Imaging was performed using a Scanora^®^ 3D-X CBCT unit (Soredex, Tuusula, Finland). Specimens were positioned upright and stabilized. Head positioning was standardized by aligning the midsagittal plane perpendicular to the floor and verifying alignment with scout views prior to exposure. Scanning was conducted using an 8 × 16 cm field of view (FOV) with manufacturer-recommended high-resolution parameters (90 kVp, 5 mA) and a 0.2 mm isotropic voxel size. Fracture status was assessed after fracture creation and before ultrasonographic evaluation by a single experienced oral and maxillofacial radiologist who did not participate in the ultrasound assessments. The reference reader was aware of the experimentally targeted fracture regions and had access to information regarding fracture generation. Fractures were defined as a visible cortical discontinuity identified on multiplanar CBCT reconstructions by the reference examiner.

### 2.5. Ultrasonographic Examination

**Figure 1:**
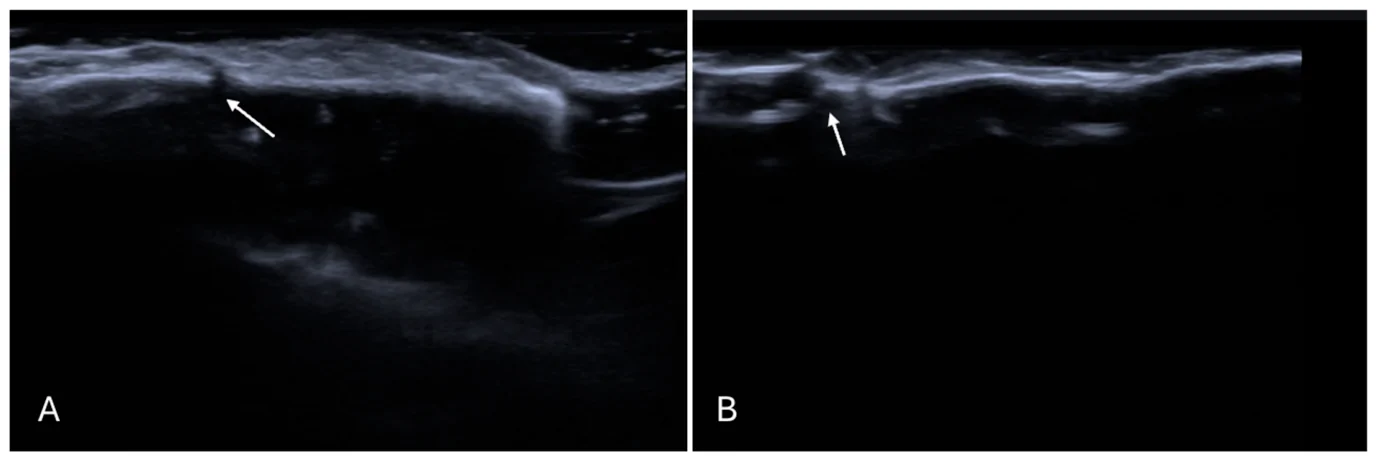
Representative ultrasonographic images of a non-displaced dentoalveolar fracture obtained with different high-frequency transducers. (**A**) Image acquired using the conventional linear transducer. (**B**) Image acquired using the hockey-stick transducer. Arrows indicate the cortical discontinuities corresponding to the fracture sites.

Ultrasonographic examinations were performed using an ACUSON Juniper ultrasound system (Siemens Healthineers, Erlangen, Germany) equipped with two high-frequency transducers: a 12L3 linear transducer (10–15 MHz) and an 18H5 hockey-stick transducer (10–18 MHz). Before each examination, a sufficient amount of ultrasound gel was applied to ensure adequate acoustic coupling. The transducer was gently positioned over the buccal soft tissues corresponding to the predetermined evaluation site while minimizing probe pressure. Gain, depth, and focal zone settings were adjusted as necessary to optimize image quality. Cortical bone integrity was assessed by evaluating the continuity of the hyperechoic cortical surface, and any interruption of the cortical line was recorded as a fracture (Figure 1). For each observer, all fracture sites were first evaluated using one transducer, after which the entire set of specimens was re-evaluated using the other transducer. The order of transducer use was randomized, and the sequence of specimens/sites was rearranged between the two evaluation sessions to minimize direct recall of previous site-specific assessments. Following each examination, the observers first recorded the presence or absence of a fracture and then rated their confidence in that assessment using a five-point Likert scale: 1, not confident at all; 2, slightly confident; 3, moderately confident; 4, confident; and 5, very confident.

### 2.6. Observer Characteristics

Two oral and maxillofacial radiologists (S.A.T. and M.M.A.) participated as independent observers. Before the evaluation sessions, both observers underwent calibration using datasets not included in the primary study sample. The calibration session was conducted three weeks before data collection to standardize interpretation criteria for both transducers. Interobserver agreement was quantified in a separate set of 20 additional fracture sites that was not included in the primary analysis. During the main study, observers were blinded to each other’s assessments. Each observer independently evaluated all fracture sites with both transducers, and no consensus assessment was used in the primary analysis. Because the physical identity of the probes was apparent during scanning, blinding to transducer type was not possible.

### 2.7. Statistical Analysis

Statistical analyses were performed using IBM SPSS Statistics for Windows, version 27.0 (IBM Corp., Armonk, NY, USA), except for the cluster-adjusted GEE analysis, which was conducted using Python (version 3.13.5) with the statsmodels package version 0.14.6. Because each fracture site was assessed with both transducers, paired binary fracture-detection outcomes were compared using the exact McNemar test. To account for the hierarchical structure of the data, an additional cluster-adjusted analysis was performed using generalized estimating equations (GEE) with a binomial distribution and logit link. Specimen was treated as the highest-level clustering variable, with fracture sites nested within specimens, and observer was included as a fixed effect. A bias-reduced robust covariance estimator was used because of the limited number of specimen clusters. A nested working correlation structure was specified to account for repeated assessments within fracture sites and clustering of fracture sites within specimens.

Observer confidence was rated on a five-point Likert scale. Because confidence scores were ordinal, within-observer comparisons between the two transducers were performed using the Wilcoxon signed-rank test. Median and interquartile range (IQR) were used as the primary descriptive measures, while mean ± SD and mean paired differences with 95% CIs were additionally reported for descriptive purposes. For paired confidence-score comparisons, Cohen’s dz was calculated as the mean of the within-pair differences divided by the standard deviation of those differences.

Interobserver agreement was evaluated using Cohen’s kappa with 95% CIs in the separate calibration dataset and, separately for each transducer, in the primary 88-site dataset. All tests were two-sided, and *p* < 0.05 was considered statistically significant.

## 3. Results

### 3.1. Fracture Detection Performance

A total of 88 experimentally created non-displaced dentoalveolar fracture sites were included in the final analysis. Observer-specific fracture-detection results for the hockey-stick and conventional linear transducers are summarized in Table 1.

Observer 1 detected 76 of 88 fracture sites (86.4%) with the hockey-stick transducer and 64 of 88 (72.7%) with the conventional linear transducer. Both transducers detected the fracture at 62 sites, whereas both failed to detect it at 10 sites. The hockey-stick transducer detected 14 fracture sites that were missed by the linear transducer, while the linear transducer detected two sites that were missed by the hockey-stick transducer. The paired difference was statistically significant (exact McNemar test, *p* = 0.004).

Observer 2 detected 74 of 88 fracture sites (84.1%) with the hockey-stick transducer and 62 of 88 (70.5%) with the conventional linear transducer. Both transducers detected the fracture at 62 sites, whereas both failed to detect it at 14 sites. The hockey-stick transducer detected 12 fracture sites that were missed by the linear transducer, while no fracture site was detected exclusively by the linear transducer. The paired difference was also statistically significant (exact McNemar test, *p* < 0.001).

In the cluster-adjusted GEE analysis, which accounted for repeated assessments within fracture sites and clustering of fracture sites within ovine heads while including observer as a fixed effect, the hockey-stick transducer was associated with higher odds of fracture detection than the conventional linear transducer (OR, 2.29; 95% CI, 1.84–2.86; *p* < 0.001).

### 3.2. Regional Fracture-Detection Rates

Regional fracture-detection rates were also examined descriptively. For Observer 1, hockey-stick versus linear transducer detection rates were 86.7% (39/45) versus 71.1% (32/45) in the maxilla and 86.0% (37/43) versus 74.4% (32/43) in the mandible; corresponding rates were 79.3% (23/29) versus 62.1% (18/29) in anterior regions and 89.8% (53/59) versus 78.0% (46/59) in posterior regions. For Observer 2, the corresponding rates were 80.0% (36/45) versus 66.7% (30/45) in the maxilla, 88.4% (38/43) versus 74.4% (32/43) in the mandible, 82.8% (24/29) versus 62.1% (18/29) in anterior regions, and 84.7% (50/59) versus 74.6% (44/59) in posterior regions. The 16 discordant sites for Observer 1 were distributed across the maxilla (*n* = 9) and mandible (*n* = 7), with 7 anterior and 9 posterior sites; the 12 discordant sites for Observer 2 were evenly distributed between the maxilla and mandible (*n* = 6 each) and between anterior and posterior regions (*n* = 6 each).

### 3.3. Observer Confidence

Observer-specific confidence scores are presented in Table 2. For Observer 1, confidence scores were higher with the hockey-stick transducer than with the conventional linear transducer (median [IQR], 4 [3–4] vs. 3 [2–3]; Wilcoxon signed-rank test, *p* < 0.001). The corresponding mean ± SD values were 3.70 ± 0.87 and 2.66 ± 0.93, respectively, with a mean paired difference of 1.05 points (95% CI, 0.78–1.31). For Observer 2, confidence scores were likewise higher with the hockey-stick transducer (median [IQR], 4 [3–4] vs. 3 [2–3]; *p* < 0.001), with corresponding mean ± SD values of 3.52 ± 0.92 and 2.70 ± 0.79 and a mean paired difference of 0.82 points (95% CI, 0.60–1.04). The paired standardized effect sizes (Cohen’s dz) were 0.849 for Observer 1 and 0.791 for Observer 2.

### 3.4. Interobserver Agreement

Interobserver agreement was assessed before the main data collection using 20 additional fracture sites that were not included in the final analysis. Cohen’s kappa was 0.828 (95% CI, 0.503–1.000), with an overall agreement rate of 95%. Following confirmation of satisfactory agreement, the observers independently evaluated the experimental fracture sites included in the final analysis. In the primary 88-site dataset, interobserver agreement for fracture detection was 84.1% with the hockey-stick transducer (κ = 0.369; 95% CI, 0.104–0.633) and 65.9% with the conventional linear transducer (κ = 0.162; 95% CI, −0.055–0.380).

## 4. Discussion

This paired ex vivo study provides experimental evidence that ultrasound transducer design can influence detection of non-displaced dentoalveolar fractures. The hockey-stick transducer detected significantly more CBCT-confirmed fractures than the conventional linear transducer and was associated with higher observer confidence. The separate pre-study calibration dataset showed high observed agreement (95%; κ = 0.828), although the relatively wide 95% CI (0.503–1.000) warrants cautious interpretation given the small calibration sample. Importantly, because the primary dataset contained fracture-positive sites only, these findings should be interpreted as a comparison of fracture detection sensitivity rather than a complete assessment of diagnostic accuracy. Because all evaluated sites were fracture-positive, the present design cannot determine whether the higher detection rate with the hockey-stick transducer reflects improved fracture visualization, a lower observer decision threshold for classifying a site as fractured, or a combination of both.

The higher fracture-detection rates observed with the hockey-stick transducer may be related to several technical and ergonomic factors. Its smaller footprint and angled design may facilitate adaptation to the complex contours of the maxillofacial region, including curved alveolar surfaces and anatomically restricted areas. In contrast, the larger footprint of a conventional linear transducer may make complete acoustic contact more difficult in some regions, potentially affecting visualization of subtle cortical discontinuities. Differences in operating frequency range between the two transducers may also have contributed to the observed findings. The hockey-stick configuration may additionally facilitate probe positioning and orientation during image acquisition. However, these explanations remain hypothetical because the present study did not independently assess the contribution of probe geometry, frequency, acoustic contact, positioning stability, or image quality. Small-footprint ultrasound transducers have also been described as useful for imaging anatomically restricted regions, including intraoral applications [18]; however, the present study evaluated both transducers only through the buccal soft tissues.

These findings are consistent with the results of the Sessiz et al. [16] study, which compared different ultrasound probes for periodontal diagnostic parameter measurements. In their prospective observational study, four operators performed real-time B-mode ultrasonography on 12 maxillary anterior teeth using an 18-MHz linear probe (extraoral) and a 15-MHz hockey-stick probe (intraoral and extraoral). They found that the hockey-stick probe demonstrated higher interrater reliability for gingival height (GH) measurements, with ICC values of 0.452 (*p* = 0.004) in extraoral application and 0.388 (*p* = 0.015) in intraoral application. Similarly, for alveolar bone crest–cementoenamel junction (ABC–CEJ) distance measurements, the hockey-stick probe showed ICC values of 0.441 (*p* = 0.033) in extraoral application and 0.461 (*p* = 0.086) in intraoral application. Importantly, Sessiz et al. [16] concluded that operator-dependent variability remains a major limitation in periodontal ultrasonography; nevertheless, the extraoral application of the hockey-stick probe provided the most consistent reliability for gingival height and ABC–CEJ measurements, supporting its potential use in real-time chairside periodontal evaluation. Although their study focused on periodontal measurements rather than fracture detection, the improved reproducibility associated with the hockey-stick transducer closely parallels our findings. Together, these findings suggest that transducer characteristics may influence both measurement reliability and fracture detection during maxillofacial ultrasonography.

Observer confidence was consistently higher with the hockey-stick transducer for both observers. Median confidence scores were 4 (IQR, 3–4) with the hockey-stick transducer and 3 (IQR, 2–3) with the conventional linear transducer for both observers, with statistically significant within-observer differences (both *p* < 0.001). The corresponding paired standardized effect sizes (Cohen’s dz) were 0.849 for Observer 1 and 0.791 for Observer 2. Previous studies have also compared the diagnostic performance of ultrasonography and CBCT in the assessment of maxillofacial fractures [19]. In the present study, the higher confidence scores obtained with the hockey-stick transducer may reflect a combination of factors, including fracture visualization, probe adaptability, ease of positioning, acoustic contact, and observer expectations related to the physical characteristics of the transducer. Although the between-transducer differences were statistically significant, the clinical relevance of an approximately one-point difference on a five-point confidence scale remains uncertain and should be interpreted cautiously. However, higher observer confidence should not be interpreted as evidence of superior overall diagnostic accuracy or as justification for replacing radiographic imaging when clinically indicated.

Previous studies have reported high sensitivity values for ultrasonographic detection of maxillofacial fractures. Nezafati et al. [4] reported a sensitivity of 94.74% for mandibular fractures, while Gadicherla et al. [15] reported a pooled sensitivity of 95% in their meta-analysis. However, these estimates are not directly comparable with the fracture-detection rates observed in the present fracture-positive experimental dataset because of substantial differences in study design, anatomical site, fracture characteristics, and reference standards. The present investigation specifically focused on non-displaced dentoalveolar fractures characterized by minimal cortical disruption, which may be inherently more difficult to visualize ultrasonographically than more conspicuous cortical discontinuities. In addition, although the ex vivo design eliminated several clinical sources of variability, the complex curvature of the dentoalveolar region and restricted acoustic access remained relevant technical challenges. Importantly, the primary purpose of this study was not to estimate the overall diagnostic accuracy of ultrasonography, but to compare fracture-detection rates and observer confidence between two transducers under the same controlled conditions. Descriptively, higher fracture-detection rates with the hockey-stick transducer were observed across maxillary, mandibular, anterior, and posterior regions for both observers; however, these regional analyses were exploratory and were not intended for formal subgroup inference.

Previous clinical studies have also explored ultrasonography for the assessment of nasal fractures [20]. From a clinical perspective, a small-footprint hockey-stick transducer may be useful for point-of-care assessment of dentoalveolar trauma, particularly when access is restricted by curved or confined anatomy. A smaller transducer footprint might also improve patient tolerance by reducing contact over painful or injured tissues; however, this potential advantage could not be evaluated in the present ex vivo model. The present results support further clinical evaluation of this probe design; they do not establish that ultrasonography can replace radiographic or CBCT assessment when clinically indicated.

Several limitations should be acknowledged. First, this was an ex vivo study using ovine specimens and therefore cannot fully reproduce vascular perfusion, edema, hemorrhage, pain, patient movement, or other features of acute human trauma. Second, the primary dataset included only CBCT-confirmed fracture sites. Consequently, specificity, false-positive rate, predictive values, and overall diagnostic accuracy could not be estimated; the principal comparison concerns fracture detection sensitivity. Third, only non-displaced dentoalveolar fractures were evaluated using a single ultrasound platform and two experienced oral and maxillofacial radiologists, which limits generalizability. Fourth, the physical differences between the probes prevented blinding to transducer type, and the observers’ awareness of the experimental fracture-positive design may have introduced performance and expectation bias. Although the two transducers were not applied in immediate succession at individual sites and the specimen/site sequence was rearranged between evaluation sessions to minimize direct recall, residual recall or anchoring bias cannot be completely excluded because the same observers evaluated the specimens with both transducers. Fifth, the pre-study calibration assessment was based on a relatively small 20-site dataset, resulting in a wide confidence interval around the κ estimate. Sixth, multiple fracture sites originated from individual ovine heads and therefore may have shared specimen-specific anatomical and bone characteristics. Although this hierarchical structure was addressed using a cluster-adjusted GEE analysis, only 15 specimen clusters were available; therefore, some uncertainty related to cluster-level inference remains. Finally, the original sample-size calculation was not based specifically on paired McNemar discordance assumptions and did not incorporate a design effect for within-specimen clustering. Therefore, the study may not have been optimally powered for the final hierarchical paired analysis. Larger prospective studies incorporating both fracture-positive and fracture-negative sites, multiple operators, and clinically encountered soft-tissue conditions are needed.

## 5. Conclusions

In this ex vivo paired comparison, the hockey-stick transducer achieved a significantly higher detection rate for CBCT-confirmed non-displaced dentoalveolar fractures and greater observer confidence than the conventional linear transducer. The observed differences may reflect the combined technical and ergonomic characteristics of the two transducers tested, rather than probe geometry alone. These findings should not be interpreted as evidence of superior overall diagnostic accuracy because non-fractured controls were not included. Prospective clinical validation is warranted before recommending one transducer as the preferred diagnostic probe for dentoalveolar trauma.

## Figures and Tables

**Table 1:** Observer-specific paired fracture-detection results for the hockey-stick and conventional linear ultrasound transducers.

Observer	Hockey-Stick Result	Linear Positive, *n*	Linear Negative, *n*	Total, *n*	Exact McNemar *p* Value
Observer 1	Positive	62	14	76	0.004
	Negative	2	10	12	
	Total	64	24	88	
Observer 2	Positive	62	12	74	<0.001
	Negative	0	14	14	
	Total	62	26	88	

Values represent paired fracture-detection assessments for each observer. For Observer 1, fracture-detection rates were 86.4% (76/88) with the hockey-stick transducer and 72.7% (64/88) with the conventional linear transducer; for Observer 2, the corresponding rates were 84.1% (74/88) and 70.5% (62/88), respectively. Within-observer paired binary outcomes were compared using the exact McNemar test. The cluster-adjusted GEE analysis incorporating both observers is reported in the Results.Values represent the number of fracture sites classified as detected or not detected by each transducer for each observer (*n* = 88 fracture sites per observer). Comparisons were performed using the exact McNemar test.

**Table 2:** Observer-specific comparison of confidence scores between the hockey-stick and conventional linear ultrasound transducers.

Observer	Hockey-Stick, Median (IQR)	Linear, Median (IQR)	Hockey-Stick, Mean ± SD	Linear, Mean ± SD	Mean Paired Difference (95% CI)	Cohen’s dz	Wilcoxon *p* Value
Observer 1	4 (3–4)	3 (2–3)	3.70 ± 0.87	2.66 ± 0.93	1.05 (0.78–1.31)	0.849	<0.001
Observer 2	4 (3–4)	3 (2–3)	3.52 ± 0.92	2.70 ± 0.79	0.82 (0.60–1.04)	0.791	<0.001

Confidence was rated on a five-point Likert scale, with higher scores indicating greater confidence. Data are presented as median (interquartile range [IQR]) and mean ± standard deviation (SD). CI, confidence interval. Comparisons were performed using the Wilcoxon signed-rank test. Cohen’s dz represents the standardized mean paired difference. Because confidence scores were ordinal, within-observer comparisons between transducers were performed using the Wilcoxon signed-rank test. Median and interquartile range (IQR) are therefore emphasized as the primary descriptive measures; mean ± SD values and mean paired differences are also provided for descriptive purposes. Cohen’s dz was calculated as the mean of the within-pair differences divided by the standard deviation of those differences.

## Data Availability

The raw data supporting the conclusions of this article will be made available by the authors on request.
